# Inhibition of *EGFR* Signaling: All Mutations Are Not Created Equal

**DOI:** 10.1371/journal.pmed.0020377

**Published:** 2005-11-29

**Authors:** Adi F Gazdar, John D Minna

## Abstract

Gazdar and Minna discuss the context and implications of a research article that examines the transformation potential and response to inhibitors of specific *EGFR* mutations found in lung cancer.

Tyrosine kinases (TKs) are found only in metazoans, where they regulate multiple critical multicellular functions, including growth, differentiation, apoptosis, adhesion, and mobility [ 1]. As these functions also play critical roles in tumorigenesis, TKs are the prototypical class of oncogenes involved in many human malignancies. Of the 90 TKs, 58 are receptor TKs grouped into 20 subfamilies, while the others are non-receptor TKs grouped into ten subfamilies.

## *EGFRs* and Cancer

**Figure 1:**
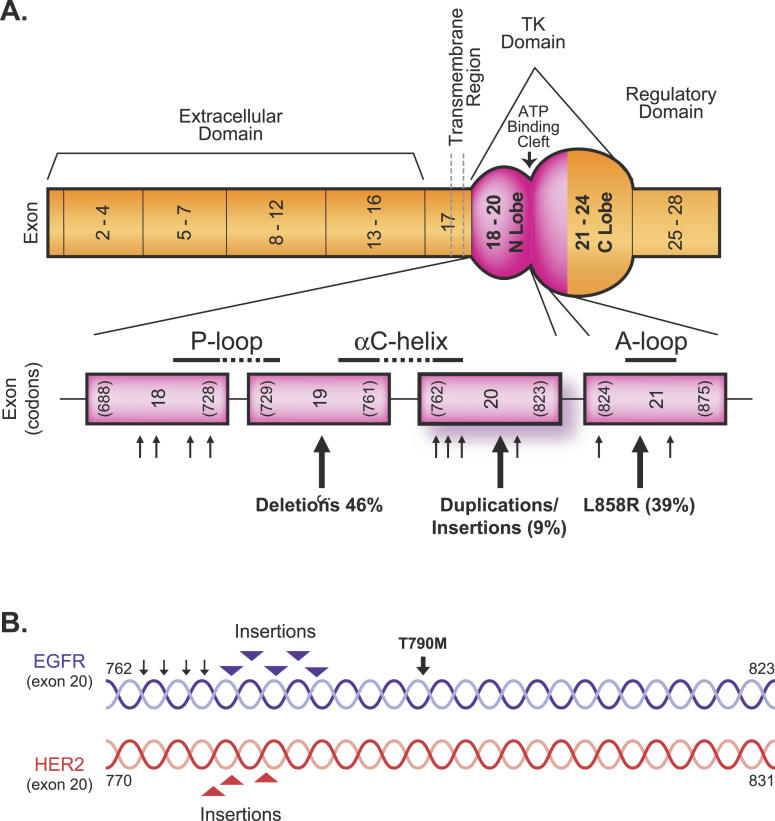
Schematic of the *EGFR* Gene with Locations of the Mutation Types (A) Mutations are limited to the first four exons (exons 18–21) of the tyrosine kinase (TK) domain encompassing all of the N lobe and part of the C lobe. The three major types of mutations, accounting for about 94% of all mutations, and their approximate frequencies are indicated by the larger arrows. The locations of most of the described rarer point mutations are indicated by smaller arrows. The mutations target regions critical for phosphorylation events (the A-loop, P-loop, and the αC helix). Data from Shigematsu and colleagues [ 8]. (B) Schematic of exon 20 of the *EGFR* and *HER2* genes, indicating the location of the described in-frame insertions/duplications (arrowheads), the T790M mutation (large arrow) and rarer point mutations (small arrows).

The *epidermal growth factor receptor (EGFR)* family is subclass I of the receptor TK superfamily, and consists of four members, *EGFR (ErbB1), HER2 (ErbB2), EGFR3 (ErbB3),* and *EGFR4 (ErbB4)*. Ligand binding results in homo- or heterodimerization and activation of the highly conserved intracellular kinase domain, resulting in phosphorylation of specific tyrosine residues that serve as docking sites of proteins whose recruitment activates a multitude of downstream signaling pathways [ 2]. Adenosine triphosphate (ATP), the phosphate donor, lodges in a cleft between the two roughly globular lobes of the TK domain. Deranged family member signaling in tumors often involves EGFR and HER2. Increased activity of EGFR signaling, often associated with adverse prognosis, has been detected in many tumors, a finding that resulted in the selection of this molecule as one of the first targets for designed therapies [ 3]. Deranged signaling may result from activating mutations, increased gene copy number, or autocrine loops. Targeted therapies that have undergone extensive clinical trials fall into two major categories: humanized forms of monoclonal antibodies that prevent ligand–receptor interaction, and small molecule inhibitors (tyrosine kinase inhibitors [TKIs]) such as erlotinib or gefitinib, which reversibly bind to the ATP binding cleft ( Figure 1A), preventing phosphorylation and subsequent downstream signaling. While antibody administration has been disappointing as a therapy for lung cancer, treatment with TKIs is associated with responses, occasionally dramatic, in highly select patient subpopulations. Less than 18 months ago, it was found that activating mutations in the TK domain of the gene occurred in a subset of non-small-cell lung cancer (NSCLC), especially adenocarcinomas, and predicted, though not precisely, the response to small molecule inhibitors [ 4–6]. In this issue of *PLoS Medicine*, Heidi Greulich and colleagues [ 7] suggest that all *EGFR* mutations are not created equal, and that different mutation classes respond differently to specific inhibitors.

## Kinase-Activating Mutations in Lung Cancer

TK domains are highly conserved and consist of two approximately globular structures, a smaller N lobe and a larger C lobe ( Figure 1A). Activating mutations in the TK domain of *EGFR* are limited to the first four exons, and show a remarkable structural diversity, including point mutations, deletions, and insertions. Mutations are largely, if not entirely, confined to NSCLC (reports of occasional mutations in other tumor types await confirmation). Two types, deletions in exon 19 and a single point mutation in exon 21, L858R ( Figure 1A), account for about 85% of all mutations [ 8]. A modest number of insertions/duplications are found in exon 20. Occasional point mutations may occur at multiple other sites and account for the remainder. Activating mutations confer ligand independence, and selectively mobilize Akt and STAT signaling pathways, which promote cell survival, but have little effect on MAP kinase regulated signaling, which induces proliferation. These mutations induce a dependency on or “addiction” to EGFR survival signals, especially when combined with allele-specific amplification, and inhibition of those signals by TKIs may contribute to the drugs' efficacy [ 9].

Some evidence existed prior to the report by Greulich et al. [ 7] that the different classes of specific mutations may vary in their clinicopathological correlations, downstream signaling events, or responsiveness to TKIs. The two major classes of mutations, deletions in exon 19 or the L858R point mutation in exon 21, may result in differential autophosphorylation of specific phosphate residues, resulting in differences in downstream signaling [ 10]. A point mutation has been described in exon 20, T790M, that is associated with resistance to TKIs (most tumors that respond to TKIs eventually recur) [ 11, 12]. Some preliminary evidence suggest that patients whose NSCLC tumors harbor the L858R mutation have a better prognosis than those with exon 19 deletions [ 8].

## Characterizing Specific Mutations

Using two in vitro model systems, an immortalized bronchial epithelial line and a mouse fibroblast line, Greulich and collaborators demonstrated the transforming abilities of mutant forms of *EGFR* (the G719S point mutation in exon 18, the L858R point mutation in exon 21, a representative deletion mutant in exon 19, and a representative insertion mutant in exon 20), but not of the wild-type *EGFR* after transfer by retroviral vector [ 7]. Transformation was accompanied by phopshorylation and activation of the appropriate downstream signaling pathways. Thus, representative mutations in all four affected exons of *EGFR* demonstrated in vitro transforming activity. However, while the wild-type form of the receptor requires ligand activation, cells with the mutant form demonstrated constitutive activation. These findings may explain why the monoclonal antibody cetuximab has little effect on lung cancer cells with activating mutations, but seems to inhibit other tumors (such as colorectal carcinomas) that overexpress wild-type *EGFR*.

While all of the mutant forms tested demonstrated transforming activity, they showed marked differences in their responses to TKIs. As previously demonstrated [ 4, 5, 12], cells with the deletion mutant and the point mutation L858R were inhibited by the TKIs gefitinib and erlotinib, while the exon 20 insertion mutation was resistant. Three patients with lung adenocarcinoma and with exon 20 deletion mutants of *EGFR* failed to show a clinical response (contrary to the expectation that, since the general response rate of tumors with *EGFR* mutations to TKIs is about 80%, two or all three of these particular ones would be responsive). However, the cells with the insertion mutation demonstrated increased sensitivity to the irreversible inhibitor CL-387,785, previously demonstrated to be active against cells with both an activating mutation and the resistance-associated point mutation T790M on exon 20 [ 11]. Thus, two very different forms of mutations in exon 20, a point mutation and an insertion, demonstrated relative resistance to the clinically widely used reversible TKIs, but exhibited sensitivity to the experimental drug Cl-387,785.

## Implications for Treatment of Lung Cancer Patients

What lessons can we learn from the report by Greulich and colleagues? First, we have confirmation that all of the recognized classes of *EGFR* TK domain mutations described in lung cancers are activating; and second, cells (and perhaps tumors) with mutations involving exon 20 demonstrate a very different pattern of response to TKIs than those harboring other mutations. There are important clinical implications from these and related findings. While CL-387,785 is not currently approved for clinical use, it or related compounds may be useful for overcoming resistance in the future. However, not all cases of resistant tumors have an identified molecular basis. Multiple other factors may modulate the response to TKIs, including deregulation of downstream pathways, amplification of target or related genes, heterodimerization with other gene family members, and autocrine loops. On a related note, mutations of *HER2* have been described in a small percentage of NSCLC tumors [ 13]. Of interest, all described mutations to date are insertion/duplications in exon 20 in a region homologous to the site of insertions in the *EGFR* gene ( Figure 1B). We predict that NSCLC cells harboring *HER2* mutations will prove resistant to the reversible TKIs, but will be sensitive to CL-387,785 (or a similar irreversible inhibitor with HER2 specificity).

The multiple mechanisms, actual or hypothetical, that can result in resistance to targeted therapies [ 14] make it unlikely that a single agent will suffice for tumor control in all cases. As stated in a recent review, “TKs are now regarded as excellent targets for cancer chemotherapy, but reality lies somewhere between the extremes of triumph and tribulation” [ 14]. Overcoming resistance by presently identified and by as yet unknown mechanisms presents many challenges to physicians, scientists, and pharmaceutical and biomedical industries.

## Abbreviations

ATP: adenosine triphosphate
EGFR: epidermal growth factor receptor
NSCLC: non-small-cell lung cancer
TK: tyrosine kinase
TKI: tyrosine kinase inhibitor
